# Post-legalization rise in German medical cannabis interest: evidence from Google trends as surrogate marker

**DOI:** 10.1186/s42238-026-00395-y

**Published:** 2026-01-27

**Authors:** Michael Constantin Kirchberger

**Affiliations:** Dermatology Center Ingolstadt, Ingolstadt, Germany

**Keywords:** Medical cannabis, Google trends, Infodemiology, Germany, Health policy

## Abstract

**Background:**

Medical cannabis legislation in Germany shifted significantly with the Medical Cannabis Act in March 2017, enabling physician prescriptions under specific conditions, and further evolved with the Cannabis Act (CanG) in April 2024, which partially decriminalized cannabis for adult non-medical use and allowed for Cannabis Social Clubs from July 2024. These reforms have prompted considerable public discussion and information-seeking. This study aimed to analyze Google search trends for medical cannabis in Germany to understand public interest nationally and regionally in response to these policy changes.

**Methods:**

Google Trends data for “medizinisches cannabis” (Germany, 2015–2025) were analyzed nationally using descriptive statistics and ITS regression for 2017/2024 policy impacts. Regional variations (16 states, 2015–2025) were assessed.

**Results:**

National search interest for “medical cannabis” markedly increased with legislative changes. Mean weekly search indices rose from 7.85 (pre-2017) to 23.79 (post-2017 Act; +203%), and further to 75.29 (post-2024 CanG; +216%). Interrupted Time Series analysis confirmed significant immediate relative increases post-2017 (135%) and post-2024 (216%), with a subsequent slight negative trend indicating attenuation after the initial peak. Significant regional disparities revealed four distinct clusters, notably Bavaria with sustained maximal interest and Bremen/Saarland showing unique temporal patterns.

**Conclusions:**

German public interest in “medical cannabis” is strongly influenced by policy, surging with the 2017 medical reform and 2024 Cannabis Act, indicating its mainstream emergence. Significant regional variations (e.g., Bavaria’s high query rates despite restrictive policies) reflect differing local contexts and implementation. Infodemiological monitoring is valuable for assessing policy response and identifying knowledge gaps.

## Background

Medical cannabis has become a prominent topic in German public health and policy over the past decade. In March 2017, the German parliament amended its narcotics laws, enabling physicians to prescribe cannabis for therapeutic use to seriously ill patients under specific conditions. This legislation, commonly referred to as the Medical Cannabis Act, took effect on March 10, 2017. It empowered physicians to prescribe cannabis flowers and extracts for patients with severe conditions for whom conventional treatments had proven ineffective or were not tolerated (Deutscher Bundestag 2017). Prior to this reform, access to medical cannabis was highly restricted; for instance, in 2016, only approximately 776 patients nationwide held special exemptions for therapeutic cannabis use.

The 2017 Act marked a fundamental shift, integrating medical cannabis into mainstream healthcare by establishing a pathway for prescriptions to be reimbursed under statutory health insurance. Subsequently, utilization increased rapidly: the number of medical cannabis prescriptions dispensed through statutory health insurance climbed from approximately 27,000 in 2017 to 95,000 in 2018 (Pharmazeutische Zeitung 2026). By 2021, annual prescriptions within this system reached approximately 372,000, corresponding to total sales of €185 million a more than four-fold increase since 2017 (Gleiss 2022).

Concurrently, Germany has initiated broader cannabis policy liberalization. In April 2023, the federal government announced a two-pillar plan to legalize cannabis for non-medical adult use. The first pillar, enacted as the Cannabis Act (CanG) of 2024, partially decriminalized the possession and home cultivation of cannabis for adults. Effective April 1, 2024, adults were legally permitted to possess up to 25 g of cannabis and cultivate up to three plants for personal use (Bundesministerium für Gesundheit 2026). The second pillar, which commenced on July 1, 2024, enabled the formation of non-profit cannabis cultivation associations (“Anbauvereinigungen” or Cannabis Social Clubs), where members can collectively cultivate cannabis for personal consumption.

These reforms represent a significant paradigm shift in German drug policy, transitioning from strict prohibition towards regulated adult access. Proponents of this change have cited public health objectives, such as disrupting illicit markets, enhancing product safety, and strengthening youth protection measures. Conversely, opponents have raised concerns regarding potential public health detriments and challenges associated with implementation.

Germany’s federal structure dictates that while the Cannabis Act is a national law, its practical implementation including aspects like the licensing of cultivation associations and the enforcement of regulations is primarily the responsibility of state (Bundesländer) authorities. These authorities have demonstrated varying degrees of preparedness and commitment to enacting the reforms, with some states like Bavaria imposing stricter administrative interpretations early on (Bayerisches Staatsministerium für Gesundheit, Pflege und Prävention 2024). Consequently, regional disparities have emerged in the pace and approach to the new cannabis policy’s rollout across the states.

Prior studies consistently demonstrate that Google search queries surge in response to significant policy events or extensive media coverage, reflecting heightened public interest or concern. In the United States internet searches for “cannabis” notably spiked during state-level ballot initiatives and elections involving cannabis legalization measures (Torgerson et al. 2020). The relative search interest for “cannabis” increased by approximately 25–30% above baseline levels during the voting months of major state legalization referenda.

In New Zealand, Google Trends data from the 2020–2021 national cannabis legalization referendum also indicated a predictive capacity for monitoring public sentiment. Search volumes during this period reportedly mirrored public engagement and foreshadowed the referendum’s outcome, particularly as specific cannabis-related search queries surged in the weeks preceding the vote, underscoring the platform’s utility in tracking public responses to contentious policy debates (Raubenheimer et al. 2020).

Further supporting these infodemiological approaches, a recent systematic review (Hallinan et al. 2023) confirmed the expanding global use of social media and search query data to investigate medical cannabis access and uptake. This review highlighted, for instance, that legislative reforms concerning medical cannabis in Europe, North America, and Australia over the past decade have generally corresponded with heightened online discussion and information-seeking behavior related to the topic.

These studies collectively underscore the utility of Google Trends as a low-cost, timely indicator of public interest in evolving drug policy issues. However, to our knowledge, no prior research has systematically examined search trends specifically for “medical cannabis” in Germany, nor has there been a comparative analysis of regional interest within Germany against the backdrop of its recent, multi-faceted cannabis policy transitions.

The foregoing literature collectively underscores the utility of Google Trends as a cost-effective and timely indicator of public engagement with evolving drug policies. Despite this, to our knowledge, a comprehensive analysis of search trends specifically for “medical cannabis” within Germany, particularly one examining both national patterns and regional variations in the context of its recent, significant cannabis policy transitions (i.e., the 2017 Medical Cannabis Act and the 2024 Cannabis Act) has not yet been undertaken.

Furthermore, the practical implementation of the Cannabis Act has revealed significant heterogeneity across federal states, with enforcement strategies ranging from facilitative to highly restrictive. This uneven regulatory landscape is accompanied by emerging clinical and epidemiological data from the early post-legalization phase in 2025. Recent nationwide analyses indicate a rise in cannabis-associated psychiatric emergencies in department settings (Eichhorn et al. 2025), underscoring the acute health challenges associated with the policy change. Concurrently, monitoring of consumption behavior suggests that while prevalence rates have not shifted abruptly, distinct patterns in user motives and sourcing are evolving (Hoch et al. 2025). Complementary projections regarding the legal versus illegal market structures further highlight the complexity of transitioning to a regulated system (Rosenkranz et al. 2025). These initial findings point to a dynamic public health environment that necessitates continuous regional monitoring.

To address this knowledge gap, the present study employed an infodemiological time-series design to analyze Google search behavior concerning “medical cannabis” in Germany from January 2015 through December 2025. This timeframe encompasses the enactment of the 2017 Medical Cannabis Act and the 2024 Cannabis Act (CanG), which partially legalized cannabis for adult non-medical use. The specific objectives of this study were: (1) to characterize national trends in public search interest for “medical cannabis” over the specified period, identifying overall trajectories and any discernible seasonal patterns; (2) to detect significant temporal change-points in search interest corresponding to these major policy interventions (March 2017 and April 2024); (3) to compare the levels and trajectories of search interest across Germany’s 16 federal states, thereby highlighting regional differences; and (4) to assess the temporal association between peaks in search interest and key cannabis policy developments or related prominent news events.

## Methods

### Data acquisition and preprocessing

Weekly Google Trends indices for the search term “medizinisches cannabis” (further referred to as medical cannabis) were retrospectively collected on January 11, 2026, utilizing the pytrends Application Programming Interface. The geographical scope was restricted to Germany, and the observation period spanned from January 1, 2015, to December 31, 2025. This specific term was selected to ensure longitudinal comparability across the pre- and post-legalization eras and to specifically isolate the dynamics of health-related information seeking from general recreational interest. Google Trends normalizes search interest data by calculating the ratio of topic-related queries to the total searching volume in the region, thereby automatically controlling population size differences. It then assigns a value of 100 to the week with peak search volume and scaling other weekly values proportionally (0–100). Two distinct analytical approaches were employed.

### National time-series analysis

The national-level search interest data were analyzed at a weekly resolution. To enhance visual interpretation of trends, a centered 12-week moving average was superimposed on the raw weekly data; however, all statistical analyses were performed on the original, unsmoothed values. The study period was a priori divided into three distinct policy epochs: (1) pre-legalization (January 1, 2015 – March 9, 2017), (2) medical-only era (March 10, 2017 – March 31, 2024), and (3) post-Cannabis Act period (April 1, 2024 – December 31, 2025). Mean search indices and standard deviations (SD) were calculated for each defined period.

Immediate changes in search interest levels between consecutive policy periods were assessed using two-sided Mann-Whitney U tests. The overall stationarity of the time-series was evaluated using the Augmented Dickey-Fuller test. To assess long-term policy impacts, an interrupted time-series regression analysis was conducted. This model regressed the weekly search index on time (weeks), incorporating two dummy variables to represent the introduction of the 2017 medical cannabis legislation and the 2024 Cannabis Act, as well as interaction terms between these dummy variables and time. Heteroskedasticity- and autocorrelation-consistent standard errors were computed using the Newey-West estimator with a lag of 12 weeks.

### State-Level heatmap analysis

To investigate regional variations in search interest, mean annual Google Trends indices for “medical cannabis” were extracted for all 16 German federal states for each calendar year from 2015 to 2025. This was achieved using the interest by region functionality of Google Trends, resulting in a state-by-year data matrix. Due to the platform’s normalization process, which recalculates indices (0–100) relative to the peak within each specific requested timeframe, absolute index values are not directly comparable across different years. Consequently, the analysis of regional differences was restricted to a purely descriptive assessment of relative search patterns rather than statistical hypothesis testing. To identify and visualize broader geographical similarities in these search interest trajectories, federal states were grouped using Ward’s hierarchical clustering method as an exploratory tool.

All statistical analyses were performed using Python version 3.12, leveraging the pandas, scipy, scikit-posthocs, scikit-learn, and statsmodels libraries. The significance level (α) was set at 0.05 for all tests.

## Results

### National search interest over time

**Fig. 1:**
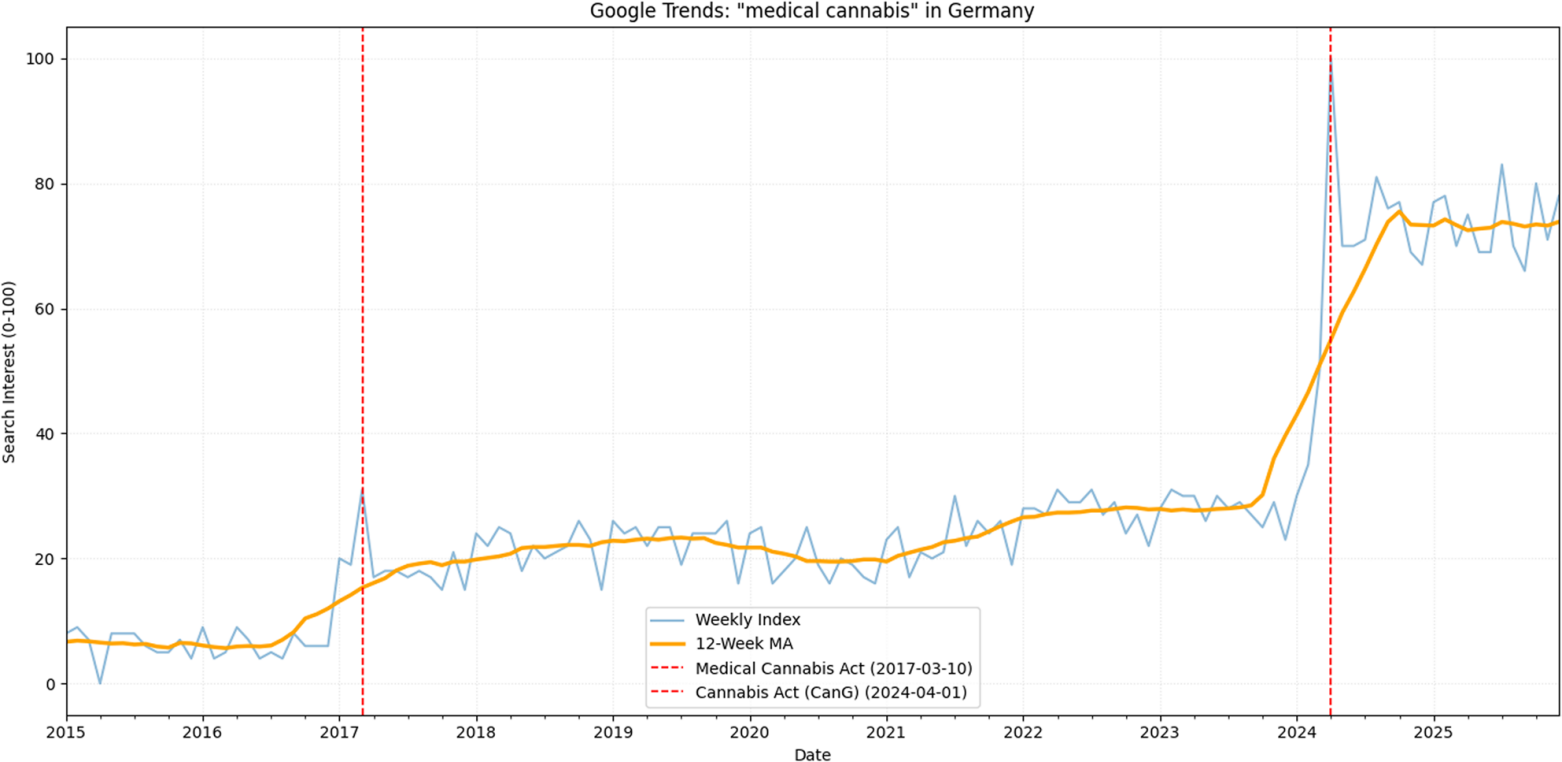
National Google Trends Search Interest for “Medical Cannabis” in Germany (2015–2025)

Analysis of Google Trends data for the search term “medical cannabis” in Germany between January 1, 2015, and December 31, 2025 (Fig. 1) reveals clear shifts in public search interest coinciding with legislative measures.

Descriptive statistics identified three distinct phases. In the pre-legalization period (January 1, 2015 – March 9, 2017), the mean weekly search interest was 7.85 (± 6.12). After the legalization of medical cannabis (effective March 10, 2017), the mean search interest significantly increased to 23.79 (± 5.57). Following the enactment of the Cannabis Act (CanG) on April 1, 2024, an even more pronounced surge was observed, with the mean weekly search index reaching 75.29 (± 8.92) for the period through December 31, 2025.

Level-shift tests (Mann-Whitney U) confirmed these observations. The increase following the 2017 medical cannabis legalization, compared to the preceding period, was highly significant (U = 112, *p* <.001). Similarly, the increase after the 2024 CanG enactment was also highly significant compared to the previous period (U = 0, *p* <.001). The time series was non-stationary according to the Augmented Dickey-Fuller test (*p* =.7897), underscoring the appropriateness of robust statistical modeling.

Interrupted Time Series (ITS) analysis, using Newey-West standard errors to correct for autocorrelation and heteroscedasticity, yielded a model with high explanatory power (R²=0.935, F(5,119) = 709.6, *p* <.001). Prior to the first intervention, there was a slight, non-significant upward baseline trend (coefficient for time = 0.3523, *p* =.071). The legalization of medical cannabis in March 2017 was associated with a significant immediate relative increase in search interest (approximately + 135%, *p* <.001) compared to the baseline level. The subsequent change in trend after this first intervention was not significant (coefficient = − 0.2074, *p* =.286), indicating a new, higher plateau of sustained interest. The enactment of the CanG in April 2024 led to another substantial immediate surge, with search interest rapidly reaching its maximum index value (100), representing an estimated relative increase of approximately + 216% (*p* <.001) compared to the prior baseline. Following this second intervention, the model showed a significant negative trend coefficient (− 1.1338, *p* <.001), indicating a subsequent attenuation of search interest after this initial peak.

### Regional variations

**Fig. 2:**
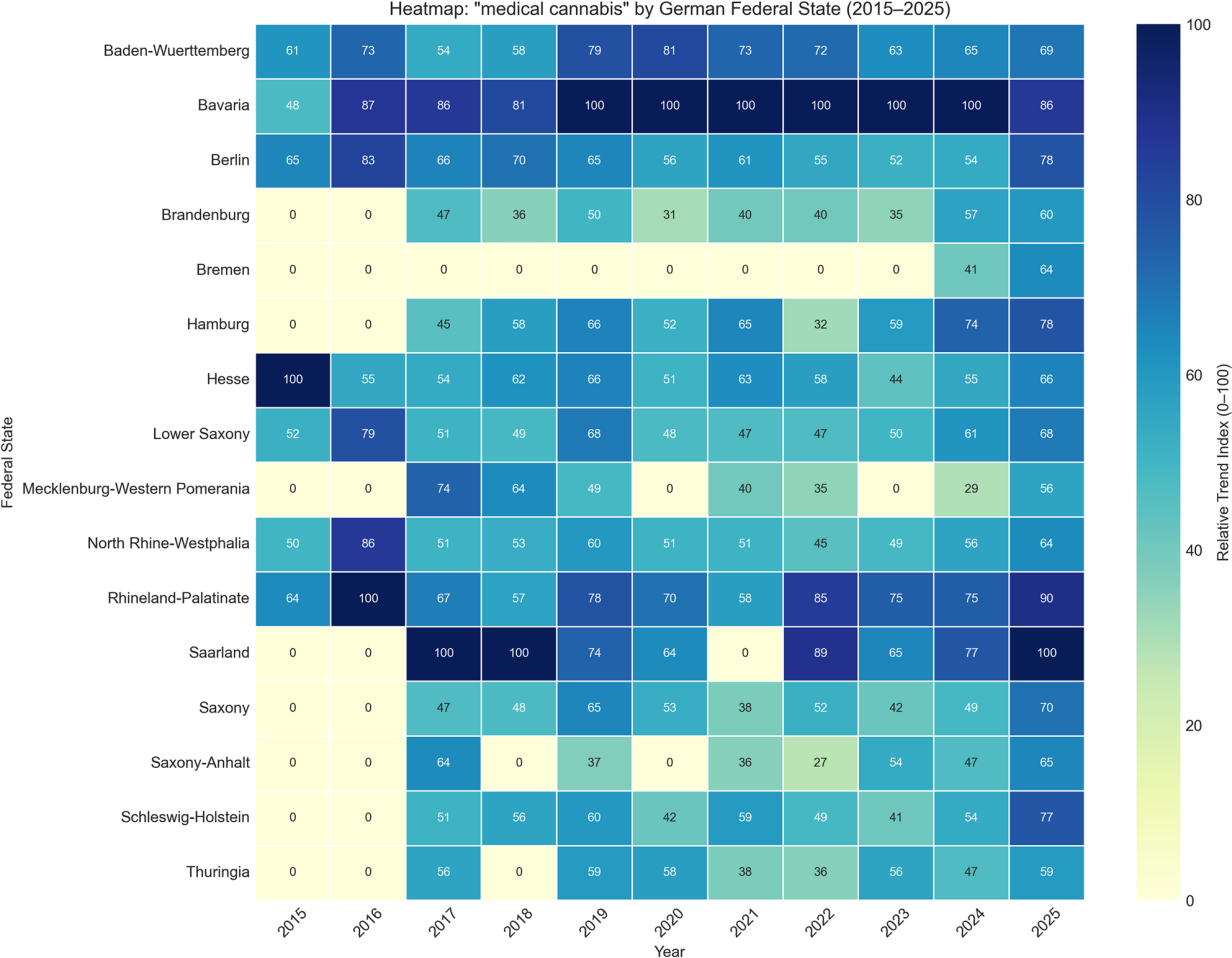
Heatmap of Relative Google Trends Search Interest for “Medical Cannabis” by German Federal State (2015–2025)

To investigate regional disparities in public engagement with the topic of “medical cannabis,” Google Trends data were analyzed across all 16 German federal states from 2015 to 2025. The relative search interest index for each state over this period is visualized as a heatmap (Fig. 2).

Given that Google Trends normalizes data relative to the peak search volume within the selected timeframe, the indices represent relative patterns within each year rather than absolute volume comparisons across years. Consequently, the analysis focuses on descriptive trends and regional trajectories.

The heatmap reveals substantial heterogeneity in search interest across the country. To explore these geographical patterns, a hierarchical clustering analysis (Ward’s method) was employed as an exploratory tool on the state-level time-series data. This descriptive grouping indicated distinct regional profiles.

Cluster 0 (e.g., Bavaria, Baden-Wuerttemberg, North Rhine-Westphalia) generally exhibited established and consistently high search interest. Bavaria is particularly notable, maintaining the maximal relative search index (100) consecutively from 2019 to 2024, indicating a sustained high level of public engagement compared to other states during these years. Hesse and Rhineland-Palatinate showed early peaks in interest (index 100 in 2015 and 2016, respectively) before aligning with the broader national trend.

Cluster 1 (primarily eastern states like Mecklenburg-Western Pomerania, Saxony, Saxony-Anhalt, but also Hamburg) typically showed lower initial interest, with several states recording indices of zero in 2015 and 2016, followed by a gradual increase.

Cluster 2 and Cluster 3 highlighted unique outliers. Bremen (Cluster 2) exhibited minimal search interest (index 0) throughout the entire period from 2015 to 2023, only registering a sharp increase in 2024 (index 41) and 2025 (index 64). Saarland (Cluster 3) displayed a highly volatile pattern, with maximal peaks (index 100) in 2017, 2018, and again in 2025, interspersed with years of lower relative interest.

A general trend of broadening interest is observable in the later years. While 2015 and 2016 show no data (index 0) for many smaller states, by 2024 and 2025, search interest is measurable across all 16 federal states, reflecting the nationwide impact of the recent Cannabis Act discussions.

Descriptive comparisons further illustrate these disparities. For instance, while Bavaria showed maximal interest (100) throughout the 2019–2024 period, states like Thuringia and Saxony-Anhalt generally remained at moderate levels (typically between 30 and 60) during the same timeframe. This persistent contrast suggests that despite uniform federal laws, the intensity of information-seeking behavior varies considerably by region.

## Discussion

### Principal findings

Our infodemiological analysis indicates that public interest in medical cannabis within Germany has been strongly event-driven, surging around key policy milestones. The Google Trends data, supported by interrupted time-series (ITS) analysis, revealed two clear step-changes in national search interest: a statistically significant, moderate rise after medical legalization in March 2017, and a more dramatic, very strong and significant rise following the enactment of the Cannabis Act (CanG) with its recreational-use provisions in April 2024. These findings imply that the German public’s information-seeking behavior regarding cannabis, even when specifically framed as “medical cannabis,” is highly responsive to major changes in law and public discourse surrounding cannabis availability. This spillover effect may be reinforced by the specific design of the German law, which prohibits commercial recreational sales. Consequently, for users unwilling to engage in home cultivation or join social clubs, the medical pathway currently represents the sole legal avenue to purchase cannabis products, potentially prompting recreational users to investigate prescriptions as a substitute. After the 2017 Act, search interest for medical cannabis increased substantially from a mean weekly index of 7.85 to 23.79, as cannabis evolved from a niche therapy to a more widely discussed option. The 2024 CanG produced an even larger response in searches for “medical cannabis,” with the mean weekly index jumping to 75.29 in the initial post-CanG period. This was unsurprising given the broader population affected and the extensive media coverage. Importantly, while ITS analysis showed a slight attenuation in trend after the initial 2024 peak (coefficient = − 1.1338, *p* <.001), overall interest remained significantly elevated, suggesting a new baseline of higher public engagement with the topic. This sustained interest could translate to more patients inquiring about medical cannabis, more clinicians needing information, and greater public dialogue on cannabis in health contexts.

Another key finding is the persistent regional heterogeneity in search interest across German federal states. Our cluster analysis grouped states into four broad patterns, though a modest silhouette score (0.306) suggests some inter-cluster similarity. Notably, states like Bavaria (Cluster 0) consistently exhibited disproportionately high interest, particularly from 2019 onwards. Saarland (Cluster 3) and Bremen (Cluster 2) showed unique trajectories warranting their own clusters, while Mecklenburg-Western Pomerania (Cluster 1) also displayed high relative interest. Conversely, Brandenburg and some other eastern German states (largely in Cluster 1) demonstrated comparatively lower search interest until the 2024 CanG spurred increases nationwide. These differences, which persisted even as all states saw interest rise post-legalization, likely stem from a complex interplay of demographics, prevailing local attitudes, media landscapes, and crucially, differences in policy implementation and enforcement.

The strong temporal correlation of search peaks with policy events, particularly the dramatic spike coinciding with the CanG’s enactment in April 2024, underscores how legislative changes can generate immediate, population-level information needs. This highlights a critical window for public health authorities to disseminate accurate information, a role partially fulfilled by the Federal Ministry of Health’s online resources concerning the Cannabis Act.

### Comparison with other nations

These results are consistent with international observations that changes in cannabis policy drive public interest spikes. Our finding of a substantial jump in searches around the 2017 medical legalization echoes the order-of-magnitude increases seen in U.S. states during their legalization votes (Torgerson et al. 2020). It also aligns with date from New Zealand showing intensified search activity preceding its 2020 cannabis referendum, where online interest peaked before the vote and was predictive of the outcome, suggesting active information seeking to inform decisions (Raubenheimer et al. 2020). While Germany’s 2024 CanG did not involve a public vote, the subsequent surge in queries reflects a similar public scramble to understand a new legal landscape. Furthermore, our study aligns with (Hallinan et al. 2023), who emphasized user-generated online data (like search queries) as valuable for understanding patient behavior and public opinion amidst evolving cannabis policies, a pattern observed globally from Australia to Canada. Germany’s experience, as reflected in our data, fits this global context, with infodemiology acting as an early indicator of policy impact on public consciousness. These scenarios underscore the infodemiological principle that when a health-related policy issue enters the public sphere, internet searches serve as a barometer of collective awareness and concern (Eysenbach 2009).

### The Bavarian case

The regional disparities in search interest, further delineated by our cluster analysis, likely mirror underlying differences in policy implementation stringency, public sentiment, and socio-cultural contexts. Bavaria’s (Cluster 0) consistently high search interest for “medical cannabis” is particularly noteworthy. Historically, Bavaria has maintained a notably stringent approach to the prosecution of cannabis-related offenses prior to the 2024 Cannabis Act; such rigorous enforcement could have plausibly increased motivation to explore or secure medical cannabis through official prescriptions, potentially perceived as a less perilous alternative in an environment with a more suppressed or riskier illicit market. This appears closely linked to the state government’s (CSU-led) vocal opposition to, and attempts to obstruct or delay, the Cannabis Act. For example, Minister-President Söder pledged restrictive implementation, and the state imposed additional requirements like mandatory prevention courses for cannabis club applicants, leading to virtually no club approvals by late 2024 (0 of ~ 37 applications). The first Bavarian clubs were only approved in early 2025 following court pressure. This restrictive approach has likely fueled public uncertainty, driving residents online for clarity on what the federal law meant for them locally. Descriptive comparisons highlight that Bavaria maintained a notably higher relative search interest compared to numerous other states, such as Brandenburg, Saxony, and Thuringia, particularly during the years 2019 through 2024.

In contrast, states like North Rhine-Westphalia (NRW) and Lower Saxony (both also in Cluster 0, indicating high overall interest) adopted more facilitative approaches to club applications, with NRW having the most applications submitted and Lower Saxony leading in early approvals. While these states also showed high search interest, it was not as consistently maximal as Bavaria’s in the years preceding the CanG. This suggests that when implementation proceeds more smoothly, some information needs might be met through official channels and local progress reports, whereas stringent implementation, as in Bavaria, may drive more prolonged and intensive self-driven online research to navigate the uncertainty.

Socio-cultural factors could also contribute. In traditionally more conservative states like Bavaria and Saarland (Cluster 3, with its unique peaks), open discussion of cannabis might have been less common historically, meaning legalization could represent a more significant societal shift, prompting more foundational information seeking. Conversely, in cities like Berlin (Cluster 0) or Hamburg (Cluster 1) with more established cannabis cultures, residents might possess greater baseline knowledge, leading to proportionately fewer new searches. Mecklenburg-Western Pomerania’s (Cluster 1) high interest, as a more rural state, could reflect a greater reliance on the internet as a primary information source, especially given it had one of the first club approvals outside the frontrunner states. The interplay seems evident: states resisting reform (e.g., Bavaria) paradoxically saw sustained high public queries, possibly fueled by concern or frustration.

### Policy implications

Our findings carry several implications for drug policy and public health strategy. First, the dramatic infodemiological response to legalization highlights a strong public demand for information during policy roll-out. Governments should anticipate these surges by proactively providing accessible, accurate resources, such as official FAQ websites (like the BMG’s cannabis portal) and optimizing them for search engines. Second, the sustained high interest post-policy presents an opportunity for continued public education on medical cannabis benefits, risks, safe usage, and the prescription process. This also implies a need for ongoing training and support for physicians who will likely face more patient inquiries. Third, the observed regional disparities, evident from the search trajectories and clustering, suggest that federal reforms alone do not ensure uniform outcomes or equitable access to information and legal pathways. Inconsistent state-level implementation, as seen with the Cannabis Social Clubs, can lead to public confusion and may undermine the Cannabis Act’s goals if residents in restrictive states remain in illicit markets. Federal guidance or minimum standards for implementation could foster greater consistency.

### Limitations

This study has several limitations. The ecological design means we analyzed aggregate search behavior, precluding individual-level inferences or causal determination, though the strong temporal correlation between policy events and search spikes is highly suggestive. Additionally, the annual normalization of regional data limits the ability to perform direct statistical comparisons of absolute search volumes across different years. Our focus on the “medical cannabis” topic captures medically oriented interest but likely underestimates total cannabis-related searching, especially around the 2024 CanG which blurred lines with recreational use in public discourse. Media coverage acts as a concurrent factor with policy events, and their effects are intertwined. Lastly, a rise in searches does not directly equate changes in knowledge, access, or health outcomes; linking these requires additional data beyond the scope of this study. The moderate silhouette score (0.306) of our cluster analysis also suggests that while the identified regional groups are indicative of patterns, they are not perfectly discrete.

## Conclusions

Public information-seeking regarding “medical cannabis” in Germany has been profoundly influenced by policy changes, as evidenced by Google Trends. Search interest dramatically increased following the 2017 medical reform and surged to unprecedented levels with the 2024 Cannabis Act (CanG), underscoring how policy interventions elevate public awareness and curiosity. This sustained, elevated interest indicates cannabis has transitioned from a niche topic to a mainstream health and social issue in Germany.

Notable regional disparities in search interest, further delineated by clustering, point to the impact of varied state-level policy implementation and local contexts. For example, Bavaria’s restrictive approach coincided with persistently high public query rates, potentially reflecting public uncertainty and a greater need for self-directed online research, suggesting inconsistent implementation can generate confusion. Effective reforms require clear, proactive public guidance, and collaborative efforts between federal and state authorities are vital for uniform information access and achieving public health and safety goals. Internet search data offers a valuable real-time lens for assessing public response to drug policy changes; incorporating infodemiological monitoring into future policy evaluations is therefore recommended to proactively identify and address public knowledge gaps.

## Data Availability

The Google Trends data used in this study are publicly available through the Google Trends website (https://trends.google.com). The specific search parameters and timeframes are detailed in the Methods section. Derived datasets generated and/or analyzed during the current study are available from the corresponding author on reasonable request.

## Abbreviations

API: Application Programming Interface
BMG: Bundesministerium für Gesundheit (Federal Ministry of Health)
CanG: Cannabis Act
ITS: Interrupted Time Series
NRW: North Rhine-Westphalia
SD: Standard Deviations
